# Bivalent oral cholera vaccination induces a memory B cell response to the *V. cholerae* O1-polysaccharide antigen in Haitian adults

**DOI:** 10.1371/journal.pntd.0007057

**Published:** 2019-01-31

**Authors:** Brie Falkard, Richelle C. Charles, Wilfredo R. Matias, Leslie M. Mayo-Smith, J. Gregory Jerome, Evan S. Offord, Peng Xu, Pavol Kováč, Edward T. Ryan, Firdausi Qadri, Molly F. Franke, Louise C. Ivers, Jason B. Harris

**Affiliations:** 1 Division of Infectious Diseases, Massachusetts General Hospital, Boston, MA, United States of America; 2 Department of Medicine, Harvard Medical School, Boston, MA, United States of America; 3 Department of Medicine, Brigham and Women’s Hospital, Boston, MA, United States of America; 4 Partners In Health, Boston, MA, United States of America; 5 NIDDK, LBC, Section on Carbohydrates, National Institutes of Health, Bethesda, MD, United States of America; 6 Department of Immunology and Infectious Diseases, Harvard T.H. Chan School of Public Health, Boston, MA, United States of America; 7 Infectious Diseases Division, icddr,b, (International Centre for Diarrhoeal Disease Research, Bangladesh), Dhaka, Bangladesh; 8 Department of Global Health & Social Medicine, Harvard Medical School, Boston, MA, United States of America; 9 Center for Global Health, Massachusetts General Hospital, Boston, MA, United States of America; 10 Division of Pediatric Global Health, Massachusetts General Hospital, Boston, MA, United States of America; 11 Department of Pediatrics, Harvard Medical School, Boston, MA, United States of America; Institut Pasteur, FRANCE

## Abstract

The bivalent killed whole-cell oral cholera vaccine (BivWC) is being increasingly used to prevent cholera. The presence of O-antigen-specific memory B cells (MBC) has been associated with protective immunity against cholera, yet MBC responses have not been evaluated after BivWC vaccination. To address this knowledge gap, we measured *V*. *cholerae* O1-antigen MBC responses following BivWC vaccination. Adults in St. Marc, Haiti, received 2 doses of the BivWC vaccine, Shanchol, two weeks apart. Participants were invited to return at days 7, 21, 44, 90, 180 and 360 after the initial vaccination. Serum antibody and MBC responses were assessed at each time-point before and following vaccination. We observed that vaccination with BivWC resulted in significant O-antigen specific MBC responses to both Ogawa and Inaba serotypes that were detected by day 21 and remained significantly elevated over baseline for up to 12 months following vaccination. The BivWC oral cholera vaccine induces durable MBC responses to the *V*. *cholerae* O1-antigen. This suggests that long-term protection observed following vaccination with BivWC could be mediated or maintained by MBC responses.

## Author summary

Oral cholera vaccines are being increasingly used throughout the world as a key component of cholera prevention programs. While several recent studies suggest oral cholera vaccines may provide durable protection, the potential mechanism that generates this long lasting immune memory and protection are unknown. Unlike antibody and antibody secreting cell responses, memory B cells are thought to be an important part of the immune responses because although these cells do not produce antibody, they are long lived and can be rapidly stimulated to produce antibodies upon re-exposure to infection. Previous studies have shown that memory B cell responses to the *Vibrio cholerae* O-antigen are associated with protection against cholera infection. In this study, we found that oral cholera vaccine generated long lasting antibody and memory B cell responses to the *Vibrio cholerae* O-antigen that remained elevated for 6 to 12 months. These findings show that oral cholera vaccination does induce a strong memory B cell response, which could play a role in the generation and maintenance of long-term protection following BivWC vaccination.

## Introduction

*Vibrio cholerae* is the causative agent of cholera and responsible for approximately 1.3 to 4 million cases of diarrhea and 21,000 to 143,000 deaths, annually[1]. Large cholera epidemics occur frequently and are even more devastating when *V*. *cholerae* is introduced into an immunologically naïve population. Oral cholera vaccines (OCVs) are an essential component of the World Health Organization (WHO) strategic roadmap that aims to reduce 90% of cholera deaths by 2030[2].

There are three currently WHO prequalified, commercially available killed whole-cell OCVs. WC-rBS (currently manufactured as Dukoral by Valneva) is a whole-cell vaccine that consists of heat and formalin inactivated *V*. *cholerae* O1 derived from both the Inaba and Ogawa serotypes and includes recombinant cholera toxin B subunit (CTB). A second bivalent vaccine, BivWC (currently manufactured as Shanchol by Shantha Biotechnics), contains *V*. *cholerae* serogroups O1 and O139 but lacks the additional CTB antigen. The third vaccine, Euvichol by EuBiologics, is considered to be a bioequivalent to Shanchol. In 2013, WHO created a stockpile of the BivWC vaccine to respond to cholera outbreaks worldwide and it has been increasingly utilized to reduce the burden of epidemic cholera[3,4].

Despite the increasing use of BivWC, there are still important questions about its immunogenicity, especially in areas outside the historically cholera-endemic areas of South Asia. While natural infection with *V*. *cholerae* induces long-term memory B cell (MBC) responses that are correlated with protection against *V*. *cholerae* infection[5], the WC-rBS vaccine does not appear to induce significant MBC responses, despite a similar initial plasma antibody response[6,7]. This finding might account for the relatively short-lived protection afforded by the WC-rBS vaccine[8]. In contrast, data from recent clinical trials and epidemiologic studies demonstrate that BivWC vaccine likely affords longer lasting protection[9]. However, there are no data currently available on whether the BivWC vaccine induces a MBC response.

To address this knowledge gap, we evaluated the development of cholera-specific MBC and serologic responses over the period of one year following BivWC vaccination in Haitian adults. The primary objective was to determine whether the BivWC vaccine, administered according to the currently recommended two-dose regimen 14 days apart, induced a MBC response in adults living in a cholera endemic region.

## Methods

### Ethics statement

The study was a conducted in the Saint Nicolas Hospital in St. Marc, Haiti, an urban center in the Artibonite Department. We invited healthy adults, ages 18–60 years presenting to the Saint Nicolas Hospital outpatient clinic between 2015–2016, to participate in this study. All participants provided a written informed consent prior to enrollment in the study. The protocol was reviewed and approved by the institutional review board of Partners HealthCare in Boston, Massachusetts and the Haitian National Bioethics Committee in Port-au-Prince, Haiti. All enrolled study participants were adults, over the age of 18 years. We excluded individuals if they had previously received OCV, were pregnant (by a urine test), or had an active gastrointestinal disorder within the 7 days prior to enrollment. We administered two doses of BivWC vaccine (Shanchol) 14 days apart to the study participants in accordance with manufacturer’s recommendations. We collected venous blood samples at day 0 (prior to vaccination), day 7 (7 days after the first dose of the vaccine), day 21 (7 days after the second vaccine dose), day 44, day 90, day 180 and day 360. We isolated peripheral blood mononuclear cells (PBMCs) by density gradient centrifugation and cryopreserved in liquid nitrogen. We also collected plasma from the processed blood after centrifugation and stored them frozen at ≥ -80°C.

### Vibriocidal antibody assay

We measured vibriocidal antibody titers at each time-point using guinea pig complement and *V*. *cholerae* O1 Ogawa PIC158 and Inaba PIC018 as the target organisms as previously described[10]. Briefly, the serum was 2-fold serially diluted in a 96 well plate 11 times (until a final dilution of 1:10,240). Each sample dilution was run in duplicate on the same plate. Diluted bacteria and complement (final concentration 1:10) were added to each well prior to incubation on a shaker at 37°C for 1 hour (50 rev/min). Subsequently, 150μl of BHI media was added per well to grow bacteria strains for 2-4hrs at 37°C until it reached an optical density (O.D.) between 0.2 to 0.28. We used a monoclonal antibody (designated OSP-A2) that binds to the O-specific polysaccharide (OSP) moiety of LPS as a standard to monitor intra-assay variability between plates[11]. The O.D.s of each sample were averaged between wells within the same plate and experiments were rejected and repeated, if we did not see 50% killing of *V*. *cholerae* with the concentration of 31.25 to 62.5ng/mL of the monoclonal antibody. All time-point samples from the same participant were run on the same plate to minimize assay variations. The vibriocidal titer was defined as reciprocal of the highest dilution resulting in >50% reduction of the optical density of control wells without plasma.

### Detection of cholera-specific antigen antibody levels in plasma

We used a standard enzyme-linked immunosorbent assay (ELISA) to measure plasma IgA, IgG and IgM antibody responses to *V*. *cholerae -*OSP as described previously[12,13]. OSP was purified and conjugated to bovine serum albumin (BSA) as previously described[14,15]. We coated ELISA plates (MaxiSorp high affinity protein binding plates) with either *V*. *cholerae* O1 Ogawa or Inaba OSP conjugated to BSA (1 μg/mL) dissolved in 50mM carbonate buffer (pH 9.6). We then incubated plates with 100 μL of plasma (diluted in 1:50) and bound antibody was detected with horseradish peroxidase-conjugated goat anti-human IgA, IgG or IgM (dilution at 1:1000) and the color substrate, ABTS/H_2_O_2_ (Sigma-Aldrich, St. Louis, MO). We measured absorbance values at 405 nm wavelength using kinetic readings (milliabsorbance/second) and SoftMax Pro software (version 5.3, Sunnyvale CA).

### Memory B cell (MBC) detection for IgG and IgA

We measured MBC responses to *V*. *cholerae-*specific antigens as previously described[16]. Briefly, we thawed and rested PBMCs overnight at 37°C, 5% CO_2_, and then resuspended to a concentration of 5 x10^5^ PBMC/ well in 24-well cell culture plates (BD Biosciences, San Jose, CA). We then added a mixture of mitogens, optimized to stimulate antigen-independent proliferation and differentiation of memory B cells into antibody-secreting cells (ASCs) to some wells, while media without mitogens was added to ‘no stimulation’ control wells. The mitogens were added to a final concentration of 3 μg of CpG (Toll-like receptor 9 agonist) oligonucleotide/mL (Operon, Huntsville, AL), 20 ng of B-cell activating factor (BAFF)/ mL (PeproTech, Rocky Hill, NJ) and 5 ng of cytokine interleukin-15 per mL (PeproTech, Rocky Hill, NJ) [17]. We then incubated the cells at 37°C with 5% CO_2_ for 6 days. For the enzyme-linked immunosorbent spot (ELISPOT) assay, we coated nitrocellulose-bottom plates (Mashan-4550; Millipore, Bedford, MA) with 100μl of *V*. *cholerae* O1 Ogawa or Inaba, OSP conjugated to BSA, at a concentration of 10 μg/ml in 1x Phosphate-buffered saline (PBS). We also coated plates with 100μl of affinity-purified goat anti-human IgG F(ab)_2_ (Jackson Immunology Research, West Grove, PA) at a concentration of 5 μg/mL in PBS (pH 7.4), CtxB at 2.5 μg/mL (Sigma Aldrich) and keyhole limpet hemocyanin (KLH, Pierce Biotechnology, Rockford, IL, 2.5 μg/mL). We used KLH as a negative control to detect non-antigen specific responses, and CtxB to assess for MBC responses that might result from exposure to natural *V*. *cholerae* infection. We used 20% of harvested PBMCs from each well to measure total IgG and IgA MBC and 80% to measure antigen-specific IgG and IgA MBCs. To detect MBCs, we used a dual color assay with horseradish peroxidase-conjugated mouse anti-human IgA (Hybridoma Reagent Laboratory, Baltimore, MA) and alkaline phosphatase-conjugated mouse anti-human IgG (Southern Biotech, Birmingham, AL). We then developed the plates with 5-bromo-4-choloro-3-indolylphosphate-nitroblue tetrazolium (BCIP/NBT, Sigma-Aldrich) and 3-amino-9-ethylcarbazole (AEC, Sigma Aldrich). Two individuals using a stereomicroscope independently quantified MBCs and the average of these determined the final number of antigen-specific and total IgG and IgA expressing MBCs per sample.

As in previous studies, we excluded from our analysis samples that did not demonstrate sufficient stimulation by mitogens or a high number of negative control spots[6,16]. Specifically, samples which did not demonstrate a ≥ 4-fold increase in total IgG or IgA antibody secreting cells after stimulation, or those in which the KLH wells contained > 3 spots were excluded from the analysis. A responder was defined as an individual with detectable MBC after vaccination with no prior MBCs on day 0 or an individual with detectable MBCs on day 0 and a 50% increase of MBCs after vaccination. The percentage of responders is the combination of both these groups over the total number of vaccinees.

### Detection of IgM antibodies from memory B cell supernatants

Because measuring IgM MBC responses by ELISPOT results in a high number of non-specific spots in both *V*. *cholerae* and KLH wells and because of the limited number of available PBMCs, we instead measured *V*. *cholerae* O1 antigen-specific IgM antibodies in MBC supernatants by ELISA as described previously[18]. We collected MBC cell culture supernatants after 6 days of incubation from both stimulated and un-stimulated cultures and added a cocktail of protease inhibitors before freezing at -80°C. We measured the OSP-specific IgM responses in the supernatants using the ELISA method described above for plasma. In addition, we measured the total IgM in each supernatant to normalize the OSP-specific IgM antibody level to the total IgM concentration per well.

### Statistical analysis

We performed statistical analyses using STATA Version 14 (StataCorp, LP, College Station, TX) and Graph-Pad Prism (Graph Pad Software, Inc., La Jolla, CA). We expressed vibriocidal titers as geometric mean titers (GMT) with 95% confidence intervals. Antibody levels were expressed as a mean of ELISA units with standard error; and antigen-specific MBC responses were presented as a percentage of the total number of IgG or IgA MBCs. We used a paired *t*-test to compare responses between baseline (pre-vaccination) and subsequent time points.

## Results

### Study enrollment and participation

**Fig 1:**
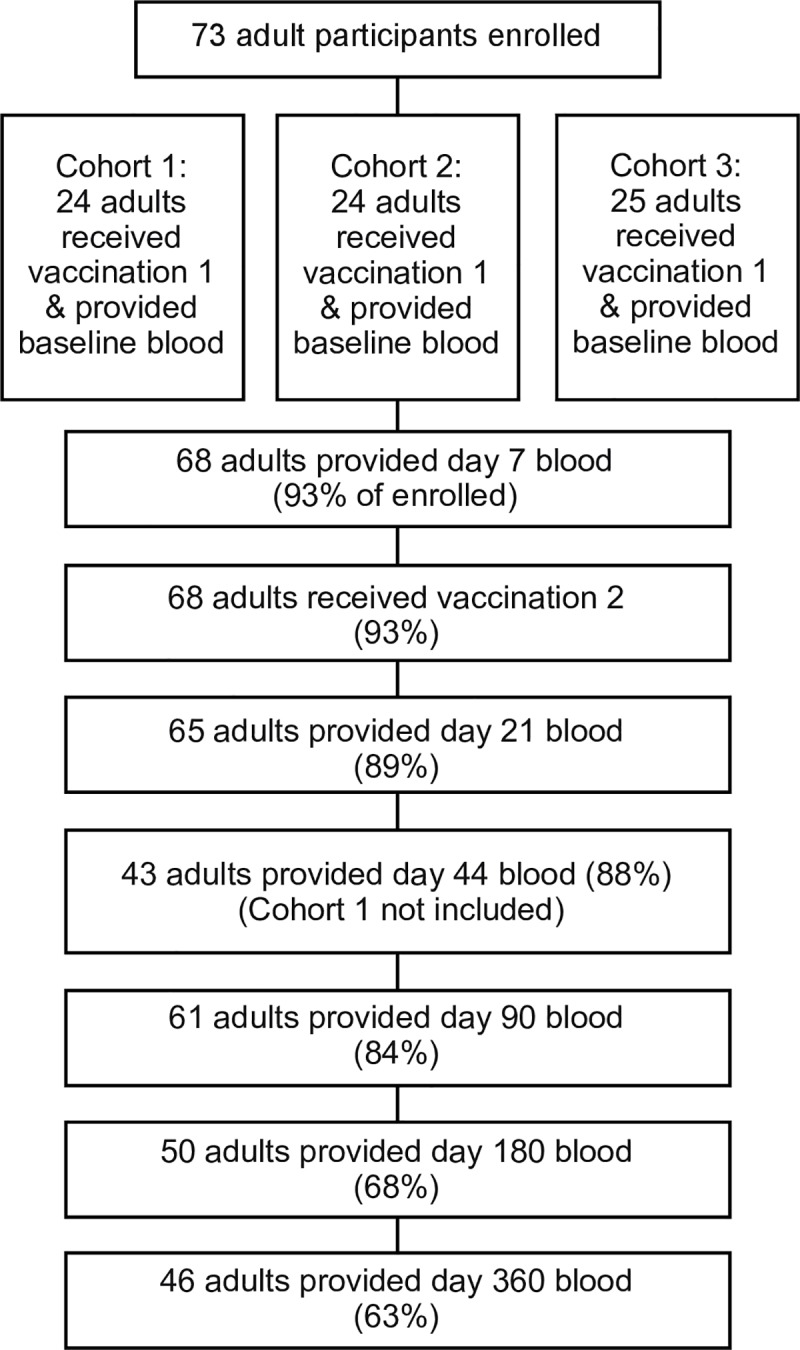
Enrollment, vaccination and follow-up of study participants.

A representation of the enrollment and follow-up of the study participants is shown in Fig 1. The participants were enrolled in three separate cohorts, and the first set of 24 participants did not have a day 44 visit which is the reason for the lower number of participants evaluated at this time point. 93% of the participants returned for a minimum of 1 follow-up point, therefore a total of 68 individuals were included in subsequent statistical comparisons. Demographic features of the study participants are listed in Table 1. No adverse events were reported related to vaccination of the study participants.

**Table 1:** Participant demographics.

Characteristics	Total *N = 73*
Age (years)	
Mean	29
Range, S. D.	18–58, 9.5
Gender (%)	
Females	32 (44)
Males	41 (56)
Blood Type (%)	
A	17 (23)
B	12 (16)
O	35 (48)
AB	9 (12)
Vibriocidal Titer at Day 0 (%)	
Ogawa ≥ 80	18 (25)
Ogawa < 80	55 (75)
Inaba ≥ 80	7 (10)
Inaba < 80	66 (90)

### Vibriocidal antibody responses

**Fig 2:**
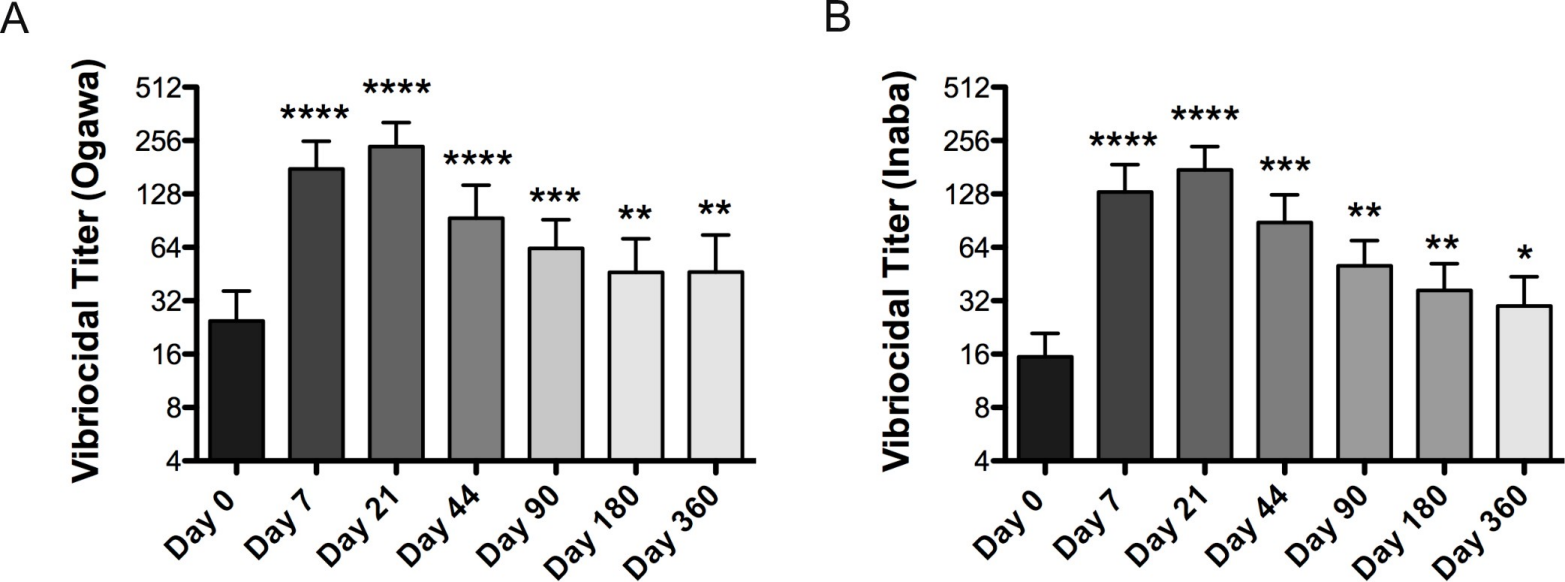
Vibriocidal responses. Geometric mean titer (±SEM) of vibriocidal responses to *V*. *cholerae* O1 Ogawa (A) and Inaba (B). Statistically significant differences relative to baseline (Day 0) are indicated. (* = P<0.05, ** = P<0.01, *** = P<0.001, **** = P<0.0001). Seroconversion for vibriocidal is defined as ≥4-fold increase over baseline. Seroconversion for OSP is defined as ≥2-fold increase in kinetic ELISA. Participants were included if they positively seroconverted by either Day 7 or 21.

Vibriocidal antibody response is a measurement of the total amount of antibodies targeting the *V*. *cholerae* bacteria and is commonly used to determine the level of prior exposure within an individual. Vibriocidal antibody responses following vaccination are shown in Fig 2, with seroconversion rates listed in Table 2. As expected, the baseline vibriocidal titers of the participants suggested a high level of previous exposure to *V*. *cholerae*; 18 of 73 (24.6%) had a vibriocidal titer ≥ 80 for Ogawa, while 7 of 73 (9.6%) had a vibriocidal titer ≥ 80 for Inaba. Consistent with our previous evaluations, over 80% of adults had a greater than fourfold increase in vibriocidal antibody titer against both serotypes following vaccination[19,20]. There is a significantly greater fold rise over baseline of Inaba vibriocidal antibody responses versus Ogawa on day 44, as measured by a paired *t*-test (p = 0.02).

**Table 2:** Seroconversion rates.

Vibriocidal (Ogawa)	83%
Vibriocidal (Inaba)	88%
OSP IgG (Ogawa)	51%
OSP IgG (Inaba)	44%
OSP IgA (Ogawa)	66%
OSP IgA (Inaba)	51%

The vibriocidal antibody response peaked on day 21 after 2 doses of vaccine and decreased in the subsequent time-points (day 44, 90, 180 and 360). However, in aggregate, the vibriocidal antibody titers remained significantly elevated over baseline titers even up to 1 year after vaccination for both the Inaba and Ogawa serotypes. Vibriocidal antibody titer geometric means and 95% confidence intervals for all time points are included in S1 Table. A small number of participants (2 for Ogawa and 1 for Inaba) also had a fourfold or greater increase in vibriocidal titers by day 360 over day 180 suggesting possible re-exposure to *V*. *cholerae* during this period.

### *V*. *cholerae*-O-polysaccharide specific antibody responses

**Fig 3:**
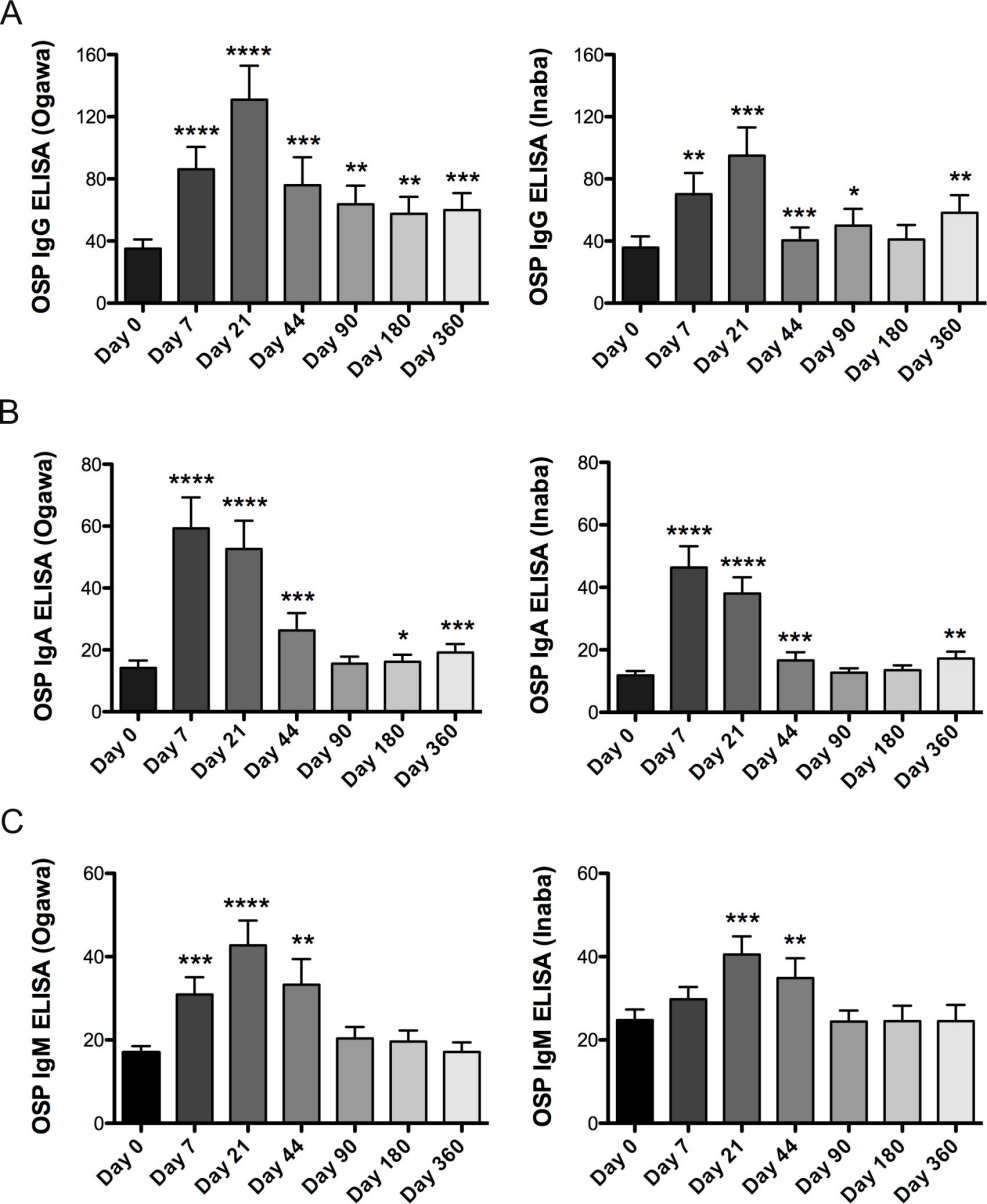
OSP-specific responses. Mean plasma antibody IgG (A), IgA (B) and IgM (C) OSP-specific responses to *V*. *cholerae* O1 Ogawa and Inaba. Statistically significant differences relative to baseline (Day 0) are indicated. (* = P<0.05, ** = P<0.01, *** = P<0.001, **** = P<0.0001).

O-specific polysaccharide (OSP) is the major antigen of immune responses that correlate to protection in *V*. *cholerae* infections. We determined antibody responses to the *V*. *cholerae* OSP antigen after each dose of BivWC vaccination and until day 360 (Fig 3). We observed a robust response increase in Ogawa-OSP IgG and remained significantly elevated over baseline up to a year after vaccination. In contrast, mean IgM responses peaked on day 21, while IgA responses peaked on day 7; and there was no clear evidence of a sustained increase in circulating IgM or IgA OSP specific antibodies beyond day 44. Similar to the vibriocidal responses, we observed an increase in responses on day 360 relative to day 180 raising the possibility of re-exposure to *V*. *cholerae* during this period.

We also performed an additional analysis, excluding a subset of individuals with evidence of recent prior exposure to *V*. *cholerae* (those individuals who had a day 0 vibriocidal titer >80). Among this subset antibody responses remained significantly elevated over baseline within the same individual. However, vaccinees with serologic evidence of recent past exposure had a significantly increased rapid Ogawa-IgG antibody response compared to individuals without evidence of prior exposure after the first vaccine dose on day 7 (p = 0.04).

We also consistently observed a lower antibody response targeting Inaba-OSP compared to Ogawa-OSP for the IgG isotype, by a paired *t*-test analysis of fold-rise antibody responses on days 21, 44, 90 and 180 (p-values of 0.02, 0.02, 0.019 and 0.003, respectively). Whereas fold-rise of IgA antibodies targeting Inaba-OSP is lower than Ogawa-OSP on day 7 (p = 0.0004). However, the differences between Ogawa and Inaba antibody increases were no longer significant when removing individuals with evidence of previous exposure.

### Memory B cell responses

**Fig 4:**
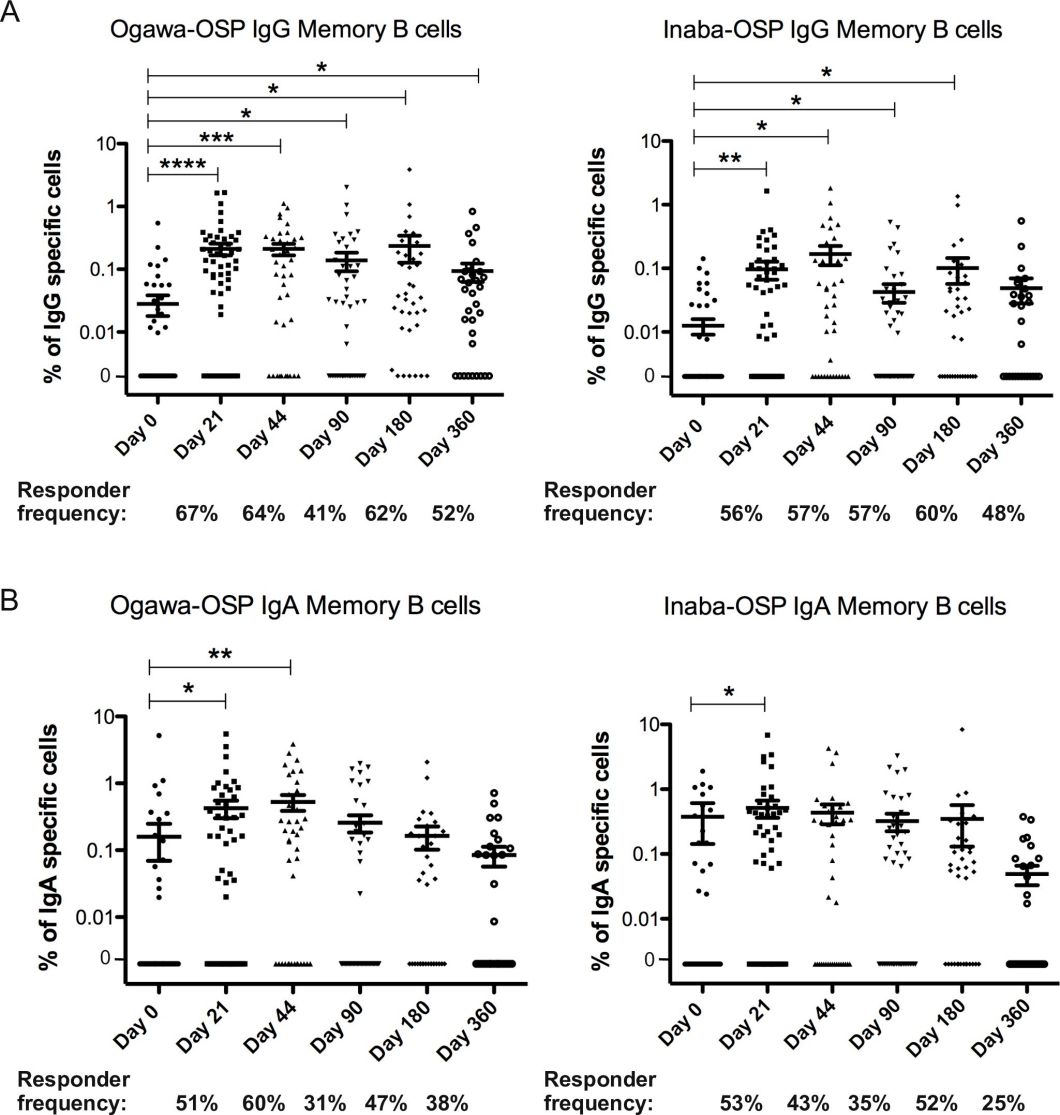
Memory B Cell OSP-specific responses. Mean antigen-specific IgG (A) and IgA (B) memory B cell responses to Ogawa and Inaba OSP, as percentages of total memory B cells, with error bars representing standard error of the mean. Statistically significant differences relative to baseline (Day 0) are indicated. (* = P<0.05, ** = P<0.01, *** = P<0.001, **** = P<0.0001).

**Fig 5:**
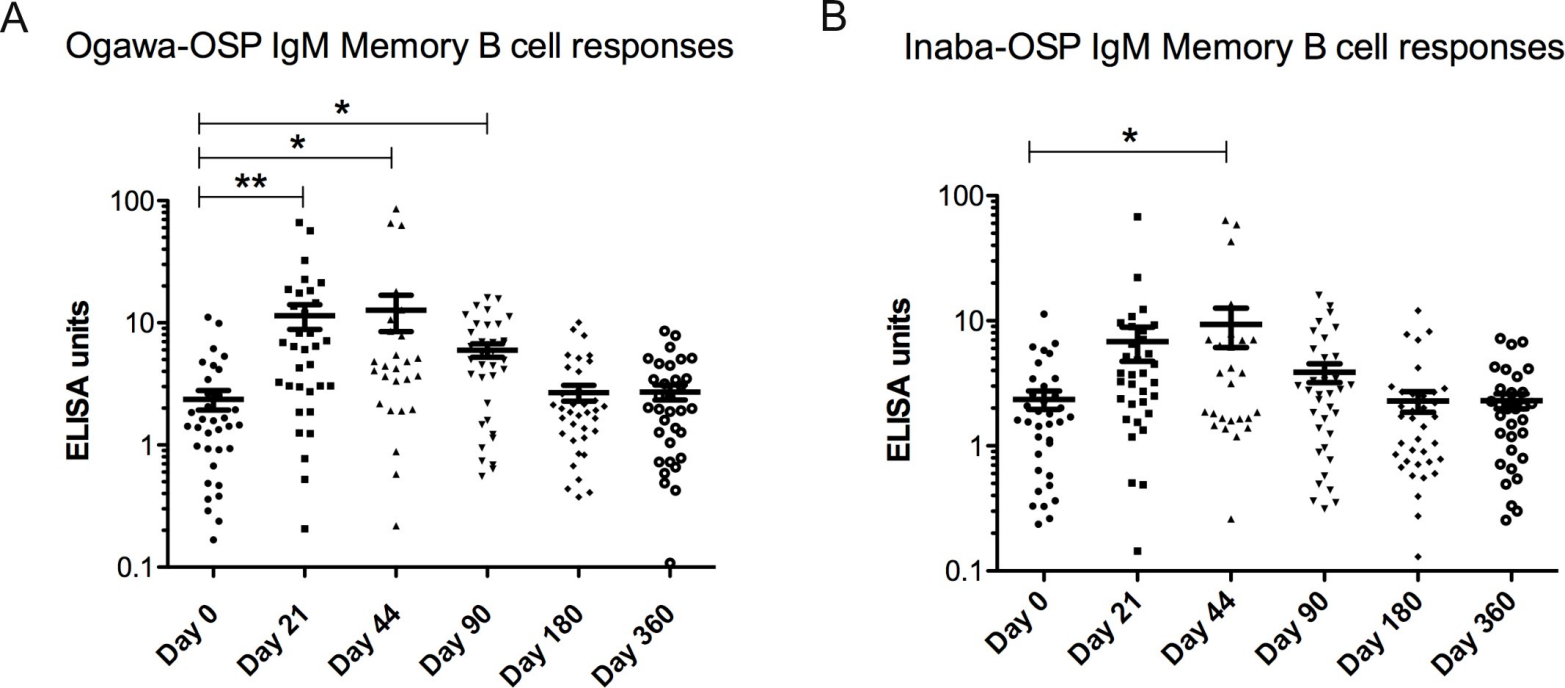
IgM Memory B Cell OSP-specific responses. Mean antigen-specific IgM memory B cell responses to Ogawa (A) and Inaba (B) OSP determined from ELISA measurements of lymphocyte culture supernatant. Statistically significant differences relative to baseline (Day 0) are indicated. (* = P<0.05, ** = P<0.01, *** = P<0.001, **** = P<0.0001).

We measured the development of MBC responses in individuals to determine whether the BivWC vaccine is capable of stimulating these memory responses (Fig 4). *V*. *cholerae* Ogawa-OSP antigen-specific MBCs demonstrated the most robust increase over baseline. The majority of vaccinees had a *V*. *cholerae* Ogawa-OSP IgG MBC response, with 67% demonstrating a response at day 21 and 64% demonstrating a response at day 44. In aggregate, these responses remained significantly elevated over baseline at 12 months post vaccination, the latest time point assessed. *V*. *cholerae* Inaba-OSP specific IgG-MBCs were also significantly elevated up to 6 months after vaccination. We also observed an increase in the number of circulating *V*. *cholerae* OSP specific IgA MBCs to both the Inaba and Ogawa serotypes, though these responses were detected in the circulation for a shorter duration of time after vaccination. Again, most vaccinees had detectable Ogawa IgA MBC responses following vaccination with a 51% responder frequency at day 21 and 60% at day 44. In addition, *V*. *cholerae* Ogawa and Inaba-OSP IgM-MBC responses (Fig 5), as measured by ELISA in cell culture supernatants, (see methods for more experimental rationale), were detected after vaccination. They were no longer elevated after day 90 for Ogawa and day 44 for Inaba.

We performed multiple regression analyses between early vibriocidal titers and subsequent immune responses to determine whether vibriocidal titers would be predictive of later immunity. However, we did not find significant associations between vibriocidal titers at baseline, day 7 or day 21 to any of the subsequent immune responses we evaluated in this study.

To evaluate whether the generation of MBC responses over the study period may have occurred from exposure to *V*. *cholerae* infection, we evaluated MBCs to the CTB protein for all individuals, at all-time points measured. CTB MBC responses were detected in 13 samples; 4% of total samples. When these samples were excluded from the overall analysis, it did not change the significance of MBC responses to vaccination at any time point over baseline.

To address the impact of likely prior exposure on memory responses after vaccination, we also performed a subsequent analysis in which we removed the individuals with baseline vibriocidal titers of 80 and above (S1 and S2 Figs). *V*. *cholerae* Ogawa-IgG MBC responses remain significantly elevated on days 21, 44 and 90, but the increase in MBCs were no longer significant for the later time points, day 180 or 360. Similarly, *V*. *cholerae* Inaba-IgG MBC responses remain significantly elevated on days 21 and 44 but are no longer significantly elevated on days 90 and 180. However, these later time points have the lowest sample numbers and the analysis could be underpowered.

## Discussion

We found that the BivWC vaccine administered using a standard two dose regimen in a cholera endemic area induced a robust MBC response to the *V*. *cholerae* O-specific polysaccharide (OSP) antigen. In addition, we found that levels of circulating Ogawa specific IgG MBC responses remained significantly increased through one year after vaccination. Our study demonstrated persistent increases in circulating vibriocidal antibody titers to both serotypes and OSP IgG antibody titers for at least one year following vaccination.

This is an important finding since OSP specific MBC responses appear to be an immunological indicator of long-term protective immunity against cholera. For example, following natural infection with *V*. *cholerae*, MBC responses remain detectable longer than other immunologic responses after a *V*. *cholerae* infection[21]; and the presence of detectable circulating IgA or IgG OSP MBCs are predictive of protection against *V*. *cholerae* following infection[5] or vaccination[22], even in exposed individuals without persistently elevated levels of circulating vibriocidal antibodies[5].

Our results differ from previous studies evaluating MBC responses to the WC-rBS vaccine [6,7,13]. These studies did not demonstrate a significant OSP MBC responses to the WC-rBS vaccination [6,7,13]. Interestingly, this difference in immunogenicity between OCVs appears to be consistent with data from both clinical trial and field effectiveness studies which demonstrate that the BivWC vaccine provides significantly longer lasting protection than the WC-rBS vaccine [9]. This difference in immunologic outcomes may reflect that CTB is a potent immunomodulatory agent. In mice, administration of CTB was found to suppress systemic immune responses, specifically dendritic cell priming Th2 responses and allergic inflammation [23,24]. CTB administered on mucosal surfaces also induces Treg cells, which would dampen immunological responses [25]. In this context, it is notable that the original trial of the precursor of the WC-rBS vaccine compared a whole cell formulation alone with a whole cell plus CTB formulation, and immunity following the CTB containing version waned more rapidly than it did for participants who were vaccinated with the whole cell only vaccine [26,27]. An alternate explanation for this difference is that it could be attributable to the antigen content of the WC-rBS vaccine versus the BivWC vaccine, as the LPS content of the BivWC vaccine is significantly greater[28].

We also observed longer lasting IgG antibody and memory B cell responses to the Ogawa-OSP antigen compared to Inaba-OSP after vaccination. Given that the Ogawa serotype has predominated in Haiti since 2010, it is possible that higher levels of previous exposure may have primed the development of IgG MBC responses. In addition, we found shorter duration of IgG MBC responses when excluding individuals with high baseline vibriocidal titers, as a measurement of recent prior exposure. These findings suggest prior exposure may lead to increased immunogenicity and longer duration of memory responses after vaccination. Future studies should take into consideration previous exposure when evaluating or comparing the immunogenicity and effectiveness of OCVs in different settings.

In conclusion, our study demonstrates the stimulation of long lasting O-specific polysaccharide antigen antibody and MBC responses by the BivWC vaccine in Haitian adults. The persistence of these immune responses was longer than expected given what has been observed in previous studies of the WC-rBS vaccine. Because MBC responses have been associated with protective immunity in previous studies [5,22], these results might provide an immunologic marker and potential mechanistic basis for the longer term protection observed following BivWC vaccination in adults living in a cholera endemic area.

## Supporting information

S1 Table Geometric means and 95% confidence intervals. Vibriocidal, antibody titer and Memory B cell geometric means and 95% confidence intervals for all time points. Units of the geometric mean and confidence interval correspond to (a) Vibriocidal titer (b) ELISA units detected in serum samples (c) percent of specific MBC cells per 1,000 total IgG or IgA cells (d) ELISA units detected in MBC culture supernatant.
(TIF)

S2 Table Immunological responses for individual vaccinees. Vaccinees de-identified, by ID number, for the individual antibody and MBC responses at each time-point measured. Legends for each individual immune response specified on the 2^nd^ tab of the spreadsheet.
(XLSX)

S1 Fig Memory B Cell OSP-specific IgG responses stratified by prior exposure. MBC responses stratified by vibriocidal titer on day 0; high prior exposure defined as 80 or above, low prior exposure as below 80. Mean antigen-specific IgG memory B cell responses to Ogawa and Inaba OSP, as percentages of total memory B cells, with error bars representing standard error of the mean. Statistically significant differences relative to baseline (Day 0) are indicated. (* = P<0.05, ** = P<0.01, *** = P<0.001, **** = P<0.0001). “N” refers to the number of samples per group.
(TIF)

S2 Fig Memory B Cell OSP-specific IgA responses stratified by prior exposure. MBC responses stratified by vibriocidal titer on day 0; high prior exposure defined as 80 or above, low prior exposure as below 80. Mean antigen-specific IgA memory B cell responses to Ogawa and Inaba OSP, as percentages of total memory B cells, with error bars representing standard error of the mean. Statistically significant differences relative to baseline (Day 0) are indicated. (* = P<0.05, ** = P<0.01, *** = P<0.001, **** = P<0.0001). “N” refers to the number of samples per group.
(TIF)
