# Supplementary figures and images for: Bivalent oral cholera vaccination induces a memory B cell response to the V. cholerae O1-polysaccharide antigen in Haitian adults
Source: PLoS Negl Trop Dis. 2019 Jan 31;13(1):e0007057. doi: 10.1371/journal.pntd.0007057 (PMC6372202; doi:10.1371/journal.pntd.0007057)

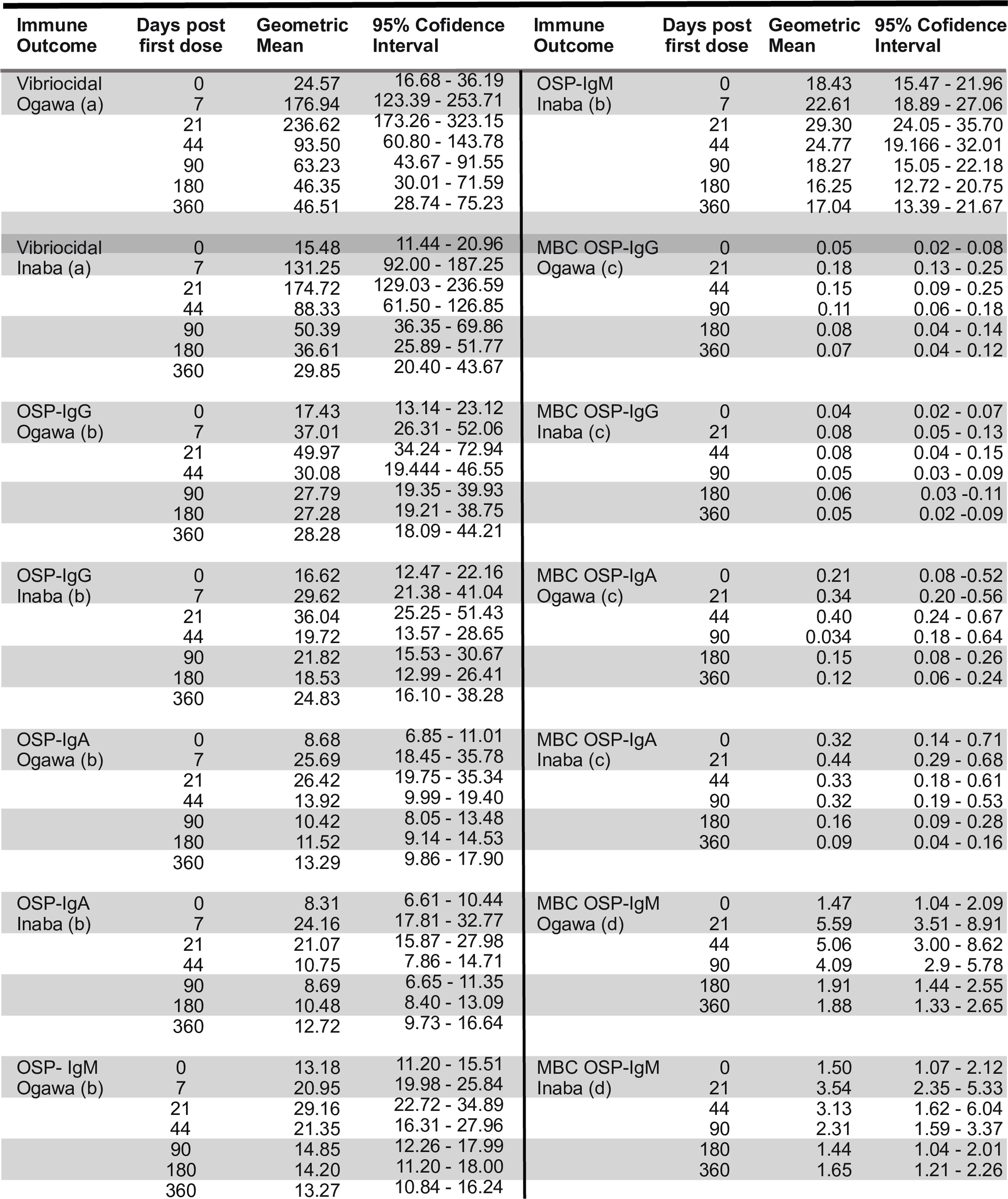

Supplement: S1 Table — Vibriocidal, antibody titer and Memory B cell geometric means and 95% confidence intervals for all time points. Units of the geometric mean and confidence interval correspond to (a) Vibriocidal titer (b) ELISA units detected in serum samples (c) percent of specific MBC cells per 1,000 total IgG or IgA cells (d) ELISA units detected in MBC culture supernatant. (TIF) [file pntd.0007057.s001.tif]

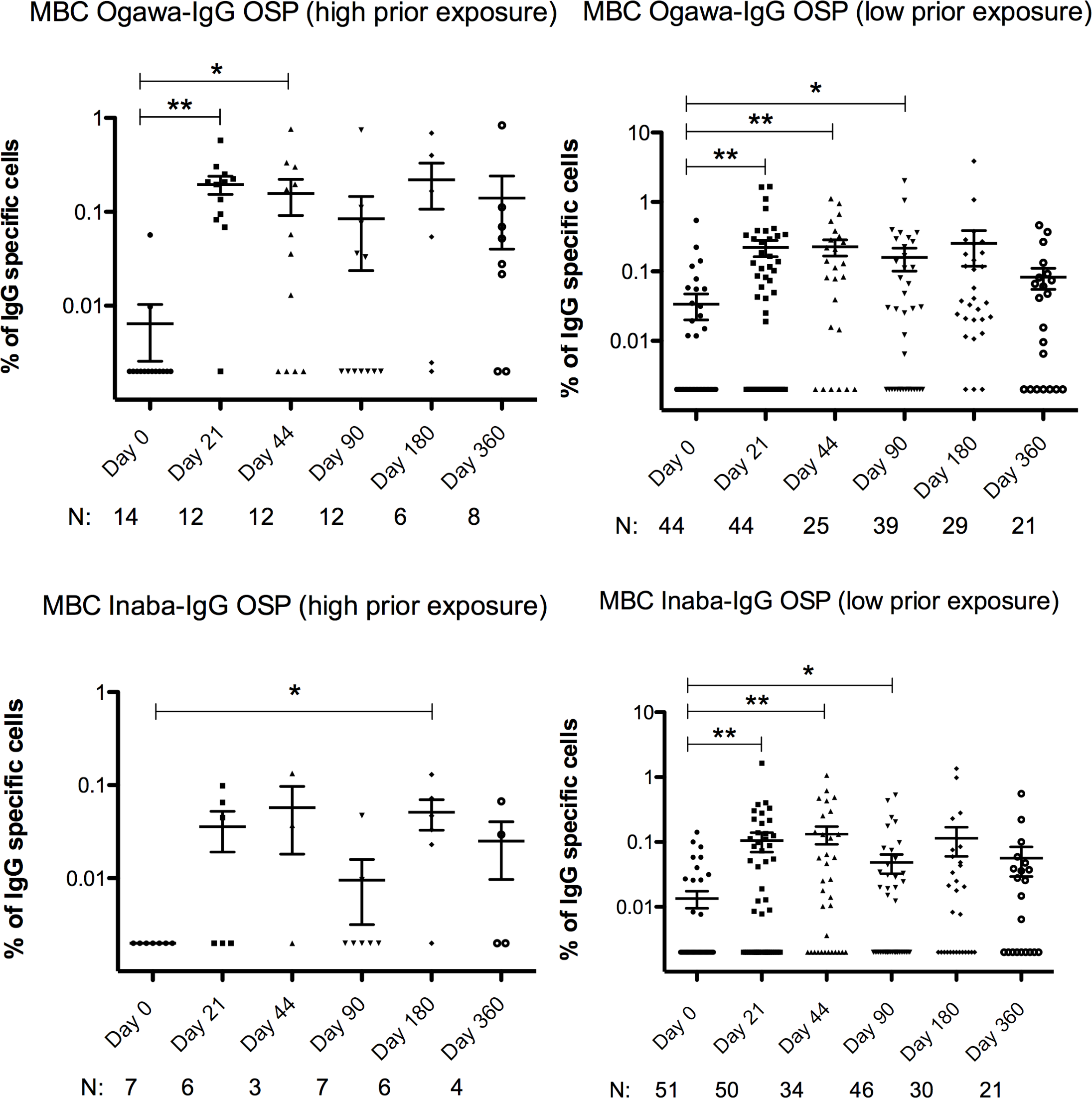

Supplement: S1 Fig — MBC responses stratified by vibriocidal titer on day 0; high prior exposure defined as 80 or above, low prior exposure as below 80. Mean antigen-specific IgG memory B cell responses to Ogawa and Inaba OSP, as percentages of total memory B cells, with error bars representing standard error of the mean. Statistically significant differences relative to baseline (Day 0) are indicated. (* = P<0.05, ** = P<0.01, *** = P<0.001, **** = P<0.0001). “N” refers to the number of samples per group. (TIF) [file pntd.0007057.s003.tif]

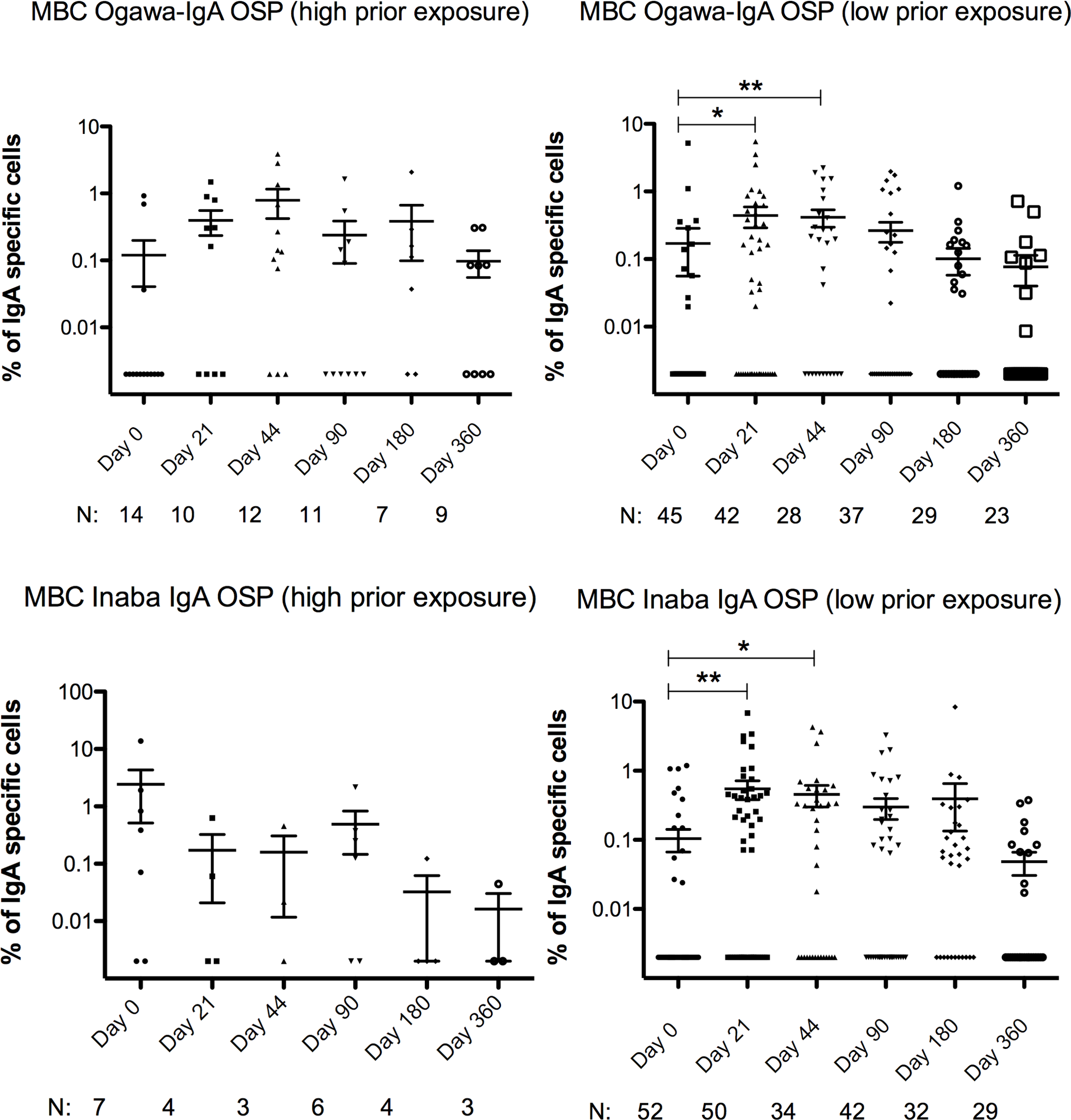

Supplement: S2 Fig — MBC responses stratified by vibriocidal titer on day 0; high prior exposure defined as 80 or above, low prior exposure as below 80. Mean antigen-specific IgA memory B cell responses to Ogawa and Inaba OSP, as percentages of total memory B cells, with error bars representing standard error of the mean. Statistically significant differences relative to baseline (Day 0) are indicated. (* = P<0.05, ** = P<0.01, *** = P<0.001, **** = P<0.0001). “N” refers to the number of samples per group. (TIF) [file pntd.0007057.s004.tif]
